# Quantifying the Sequence-Dependent Kinetic Protection of Reduced Heme in Peptide Amphiphile Nanofibers

**DOI:** 10.1021/acs.jpcb.6c02307

**Published:** 2026-08-10

**Authors:** Blake Campbell, Abigail Rogers, Nathan A. Hernandez-Martinez, Subas Dangi, Lee A. Solomon

**Affiliations:** † Department of Biology, 3298 George Mason University, Fairfax, Virginia 22030, United States; ‡ Department of Chemistry, George Mason University, Fairfax, Virginia 22030, United States

## Abstract

Replicating the protective dielectric environment of natural cytochromes within synthetic assemblies is essential for the development of air-stable bioelectronics. We report a redox-triggered “supramolecular gate” in self-assembled peptide amphiphile (PA) nanofibers that provide significant kinetic protection to ferrous heme B under aerobic conditions. By systematically varying side-chain steric bulk and hydrophobicity in a series of c16HHX4K3 PAs, we identified a phenylalanine variant (c16HHFL3K3) that extends the ferrous lifetime to over five seconds. Using a comprehensive multicomponent kinetic model, we resolve an initial protection phase in the c16HHFL3K3 variant that is absent in smaller side-chain controls like alanine. Potentiometric titrations revealed massive redox hysteresis (ΔE_m_ up to 305 mV), representing the mechanistic barrier required for the transition to a “locked” state. Circular dichroism spectra support this transition as a cooperative increase in superhelical twisting upon reduction. These findings establish sequence-specific design rules for stimulus-responsive “insulation” in peptide-based materials, enabling the protection of reactive redox centers in atmospheric environments.

## Graphical abstract

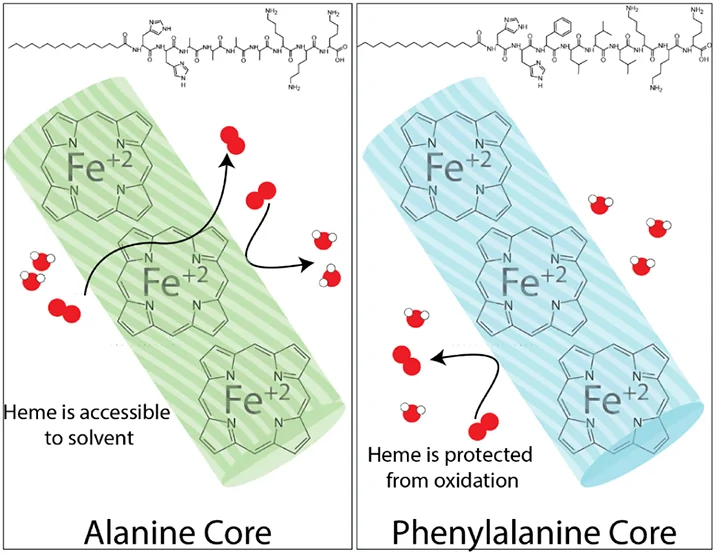

## Introduction

The fundamental mechanisms by which Nature stabilizes reactive redox centers in their reduced state, such as the ferrous Fe^2+^ state of heme B, remain a central focus of bioinorganic and materials chemistry. In biological systems, the transition of heme from the ferric to the ferrous state is a requisite for directional electron transfer and long-range electron transport (LRET).^1^ Replicating this behavior without electron leaks and reactive oxygen species (ROS) generation in synthetic and biobased material systems is paramount for the development of sustainable bioelectronics, microbial fuel cells, and artificial oxygen carriers.^2–5^

This challenge is central to understanding how LRET occurs in synthetic conductive polymers and peptide-based nanowires.^6–8^ It is difficult to use these materials as model systems to investigate how changes to the protein around the redox centers control electron transfer. Indeed, many synthetic LRET materials must be used in anaerobic chambers or under high-vacuum conditions to maintain functionality.^9^ In biological nanowires, the protein matrix acts as a dielectric barrier that tunes the reorganization energy (λ) and shields the cofactors from solvent-mediated quenching.^10,11^ Without this precise control over the local dielectric environment, electrons are lost to ambient O_2_, generating reactive oxygen species (ROS) that degrade the scaffold.^12,13^ We therefore focus on how the peptide sequence modulates this insulation to maintain the ferrous state under aerobic conditions.

Natural multiheme proteins that function as cytochrome nanowires rely on different structural features to protect the heme cofactors and prevent the “leakage” of electrons to the environment. For example, OmcS uses various parts of the protein to cover or restrict solvent access to its six c-type heme cofactors.^1^ Another technique, used by the MtrC protein, is to have a separate subunit that contains a CX8C motif that inhibits the formation of ROS from the complex.^14^ However, the OmcZ protein, which has a higher conductivity than OmcS, has slightly more solvent accessible cofactors to drive down the redox potential.^15^ Together, these examples show that protection of the cofactor is important, but a balance with functional needs must also be considered. However, we could not find any published articles that directly study how much insulation is needed for LRET in biological or peptide-based systems nor, provide a model system in which to test this parameter.

Natural globins, such as myoglobin, minimize electron leakage through an exquisitely tuned heme-binding site where steric bulk and specific residues (e.g., the distal histidine) create a high-energy barrier to the oxidation of the heme cofactor by bound oxygen.^16^ However, these folded systems often lack the modularity and large-scale assembly properties required for materials science. More modular or diverse *de novo* protein design has successfully produced helical bundles and folded scaffolds capable of tuning the midpoint potential E_M_ of heme,^17–21^ however, these too were not designed for material synthesis, and are not suitable as model systems to study OmcZ and MtrABC

Peptide amphiphiles (PAs) are molecules that consist of a hydrophobic alkyl tail conjugated to a β-sheet-forming peptide headgroup.^22,23^ They offer a unique alternative to monomeric proteins that function as biological-based materials that can be modified to carry natural or synthetic redox-active cofactors.^24,25^ PAs self-assemble into nanofibers that provide a suitable and modular environment for cofactor coordination.^26^ Unlike the rigid, predefined folds of globular proteins, PA fibers are semicrystalline and potentially permeable to small molecules like O_2_.

Different sections of the peptide sequence play different roles (Figure 1).^22,25,27^ The lysine residues at the head form an environmentally responsive region that promotes polymerization when they are neutralized. The leucine residues form the structural region, which promotes the β-sheet structure and can be modified to change the cofactor environment. The histidine residues make up the functional region, named for their heme-binding function. Finally, the lipid tail forms the hydrophobic core, which imposes directional order and structural nucleation on the system.

**Figure 1:**
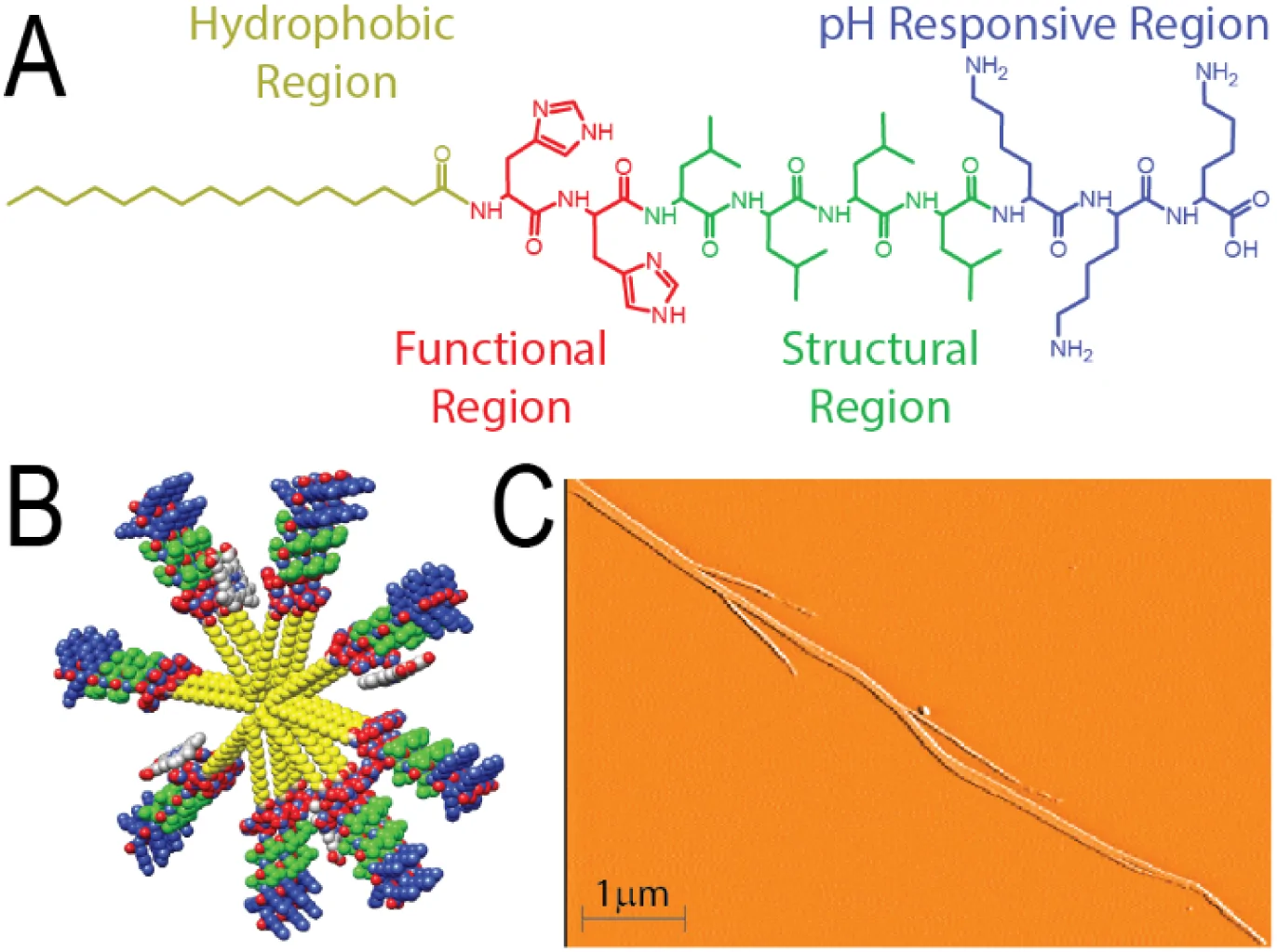
(A) Chemical structure of the PA c16HHLLLLKKK (L4) seen in this paper. (B) Model of a cross section and (C) AFM image of the L4 PA.

The dynamic nature of the supramolecular scaffold presents challenges: while it allows for modularity, it often leads to “leaky” structures where the core is insufficiently shielded. Our past work with PA fibers has focused on tuning the redox properties of bound heme cofactors. Dutta et al. showed that sequence length in the structural region did not have a significant effect on the Reduction Potential (E_M_),^27^ but different loading ratios were able to shift the E_M_ over 100 mV. This work did not focus on the kinetic stability of the reduced state in aerobic conditions, thus the structural response of the fiber to the heme’s redox state, is largely unexplored. There is a critical need to understand how the primary sequence of the peptide monomer dictates the physical permeability of the resulting nanofiber shell.

We hypothesized that the supramolecular assembly undergoes a redox-state-dependent conformational change that modulates the permeability of the peptide shell to exogenous oxidants. This observation posits that PA assembly is not a static tube but rather a responsive material that can actively change its structure in response to the oxidation state of bound heme cofactors. Such a mechanism would mimic the conformational protection seen in natural globin proteins,^28,29^ but would be driven by the collective interactions of a self-assembling PA material. By shifting the focus from static shielding to dynamic responsiveness, we sought to determine if simple, 9-residue peptide plus palmitic acid tail PAs could be programmed to protect their electronic payloads through cooperative structural reorganization.

In this study, we systematically varied the amino acid sequence within the core of a bis-histidine coordinating PA c16HHX4K3 to probe the relationship between side-chain hydrophobicity, supramolecular order, and oxidation kinetics. Specifically, we investigated the role of aromatic bulk. We chose phenylalanine as a key variable due to its ability to participate in π-stacking and its significant steric volume, used to protect ferrous heme B.^30–32^ By integrating stopped-flow spectrophotometry with an 8-parameter kinetic model, we map the transport of oxygen through the fiber. Furthermore, through the observation of significant redox hysteresis and reduction-induced circular dichroism (CD) changes, we demonstrate that the most stable variants utilize a combination of steric bulk and hydrophobicity to achieve ferrous lifetimes previously unseen in synthetic peptide systems.

## Materials and Methods

### Materials

All reagents and Fmoc-protected amino acids for peptide synthesis were purchased from Sigma-Aldrich (St. Louis, MO, USA) and used without further purification unless otherwise noted. Hemin chloride (Heme B) was stored at 4 °C in the dark. All aqueous buffers and solutions were prepared using Milli-Q water.

### Peptide Synthesis and Purification

Peptide amphiphiles (PAs) were synthesized via automated solid-phase peptide synthesis (SPPS) using a PurePep Chorus synthesizer (Gyros Protein Technologies). Synthesis was performed on a 0.1 mmol scale using Rink amide MBHA resin. Fmoc-deprotection was achieved using 20% piperidine in dimethylformamide (DMF). Amino acid coupling was performed at 60 °C for 8 min using 3 equiv of HCTU, with the exception of Fmoc-His­(Trt)-OH, which was coupled at room temperature to prevent racemization. Palmitic acid was conjugated to the N-terminus using the same coupling conditions.

The PAs were cleaved from the resin using a cocktail of trifluoroacetic acid (TFA)/triisopropylsilane (TIPS)/water (95:2.5:2.5 v/v) for 2 h. Crude peptides were precipitated in cold diethyl ether and purified by semipreparative reverse-phase HPLC (Phenomenex C18ec column) using a linear gradient of water and acetonitrile (ACN) containing 0.1% TFA. Purity was confirmed to be >95% by analytical HPLC, and molecular weights were verified via MALDI-TOF mass spectrometry (Figure S1, S2).

### Sample Preparation

Stock solutions of PAs (10 mg/mL) were prepared in deionized water. Hemin chloride stocks (15.3 mM) were prepared in anhydrous DMSO and shielded from light to prevent photodegradation. For all biophysical experiments, the PA and heme were mixed at a 6:1 molar ratio to ensure complete heme sequestration. To promote thermodynamic assembly, samples were incubated at 90 °C for 10 min and slowly cooled to room temperature prior to measurement.

### Stopped-Flow Spectrophotometry

Oxidation kinetics were monitored using an OLIS RSM 100 stopped-flow spectrophotometer equipped with a rapid-scanning monochromator. Reduced samples were prepared at pH 11 (15 mM NH_4_OH) with 50 μM heme and 300 μM PA. The samples were prereduced using a substoichiometric concentration of sodium dithionite and rapidly mixed with oxygen-saturated buffer at 15 °C. Data were acquired at 1000 scans/s for the initial 4 s to capture rapid phase kinetics, followed by a 60 s acquisition at 61 scans/s. Kinetic traces were analyzed using the program Mathematica. Data were baseline-corrected before being plotted for fitting. Raw spectra can be seen in Figure S3.

### Potentiometric Redox Titrations

Midpoint reduction potentials (E_M_ values) were determined via spectroelectrochemical titrations as previously described by Dutton et al.^33^ Samples containing 25 μM heme and 150 μM PA in 125 mM NH_4_OH were titrated with sodium dithionite (reductant) and potassium ferricyanide (oxidant) in the presence of a redox mediator cocktail (1 μM final concentration each: phenazine methosulfate, gallocyanine, indigo carmine disulfate, 2-hydroxy-1,4-naphthoquinone, anthraquinone-2-sulfonate, benzyl viologen, methyl viologen, and anthraquinone). Solution potential was monitored using a platinum 3 M Ag/AgCl combination electrode (Microelectrodes, Inc.) calibrated against quinhydrone standards. Potentials were corrected to the standard hydrogen electrode (SHE). UV–vis spectra were recorded after each potential equilibration (5–10 min). For all redox titrations, we started at the fully oxidized state (∼100 mV), and fully reduced the sample. We then reoxidized the sample and rereduced it so that each titration ran in three directions. The fraction of reduced heme was determined by the intensity of the α-band (556 nm) and fitted to the Nernst equation:

1 $$E_{h}=E_{m}+\frac{RT}{nF}\ln\left(\frac{\left[Ox\right]}{\left[Red\right]}\right)$$

where E_h_ is the environmental potential, R is the gas constant, T is the temperature in Kelvin, n is the number of electrons, and F is Faraday’s constant. Fits were done using the program Mathematica.

### Atomic Force Microscopy (AFM)

Fiber morphology was visualized using a Nanosurf CoreAFM. Samples (10 μM peptide) were prepared at pH 7 (10 mM HEPES) or pH 11 (125 mM NH_4_OH), drop-cast onto freshly cleaved mica, and air-dried. Imaging was performed in dynamic force mode using contact mode cantilevers (force constant: 48 N/m; resonance frequency: 75 kHz).

### Circular Dichroism (CD) Spectroscopy

Secondary structural characterization was performed on a Jasco J-1100 CD spectrophotometer using a 1 mm path length quartz cuvette. Spectra were acquired at 20 °C from 190 to 250 at 0.2 nm intervals. Samples were prepared at 150 μM PA (with or without 25 μM heme) at pH 11. Each spectrum represents the average of three accumulations with a scanning speed of 50 nm/min.

## Results

### Supramolecular Assembly and Design Rationale

The primary objective of this study was to investigate how variations in the supramolecular peptide core influence the oxidation kinetics of bound heme B. We first wanted to investigate the effect of bis-his ligation compared to mono-His, which was the exclusive ligation in our previous paper, and does not promote bis-his heme ligation.^24,27^ We hypothesized that substituting the alanine residue with a second histidine would further protect the ferrous heme from oxygen exposure by enabling histidine residues from adjacent peptides to simultaneously coordinate a single heme molecule. Stopped-flow analysis supports this hypothesis. Figure 2 shows both the His-Ala and His-His peptide being exposed to an oxygen-saturated buffer. We observed that the PA fiber of c16AHL4K3 (L4HA) has a less stable reduced heme B compared to the c16HHL4K3 (L4HH) peptide at pH 11. L4HA is entirely oxidized by approximately one second, whereas L4HH remains partially reduced throughout the 3.5- second time frame shown here. Subsequent PA designs, therefore, incorporate this bis-histidine ligation.

**Figure 2:**
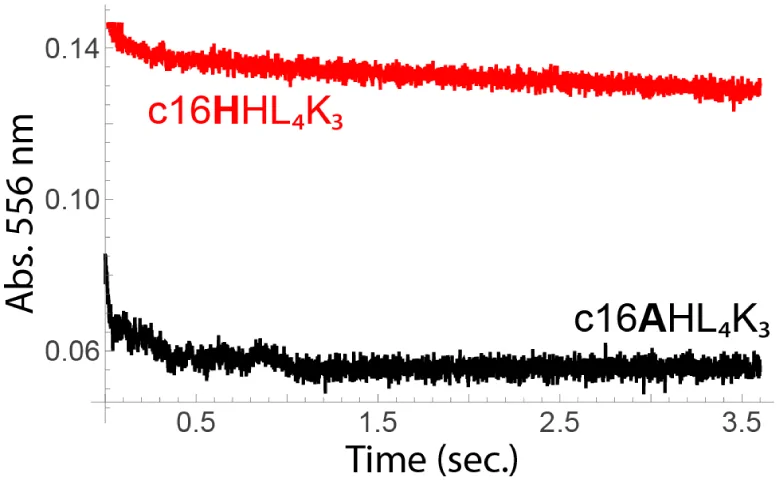
Comparison of the oxidation kinetics of the PA’s c16AHLLLLKKK and c16HHLLLLKKK. All samples were prepared with 50 μM heme and 300 μM PA. Temperature was set to 15 °C, samples are in 15 mM NH_4_OH, pH 11.

We next modified the peptide sequence to probe side chain size and hydrophobicity. All of the peptides in this work share a general c_16_HHX_4_K_3_ architecture, where the core four residues (X_4_) were systematically varied to tune secondary structure and the local chemical environment around the heme (Figure 3). We kept the peptide c16HHL_4_K_3_ as our base peptide, and made additional modifications to it to test our hypotheses. Observing that it remains reduced for at least 3.5 s when exposed to oxygen, we felt that modifications would illuminate how that protection works. c_16_HHA_4_K_3_ (A4) and c_16_HHF_4_K_3_ (F4) were designed to determine the effects of side chain size and the effects of steric bulk throughout the peptide structural region. Phenylalanine was chosen for this due to its large size and lack of hydrogen bond donors/acceptors. Alanine was chosen due to its small size, which we hypothesized would allow more water into the core, and serve as a negative control for protection. c_16_HHFL3K3 (L3F) and c_16_HHYL3K3 (L3Y) were designed to increase the steric bulk slightly around the heme and test the side chain hydrophobicity by adding the additional hydroxyl group on the side chain. We chose to only replace one Leu residue with Phe because replacing all four would add too much steric bulk to the structural region, which is not the goal of this peptide. A single Phe mutation allowed for slightly more hydrophobic character in this region, while preserving the Leu packing. Tyr was used in the complementary peptide L3Y to remove some of the hydrophobicity by adding that hydroxyl group, while keeping the structural elements of the Phe residue. This served as a negative control for this hypothesis. Along with the central L4 peptide, we now had five peptides that we could use to test how peptide sequence affects the lifetime of reduced heme when exposed to oxygen.

**Figure 3:**
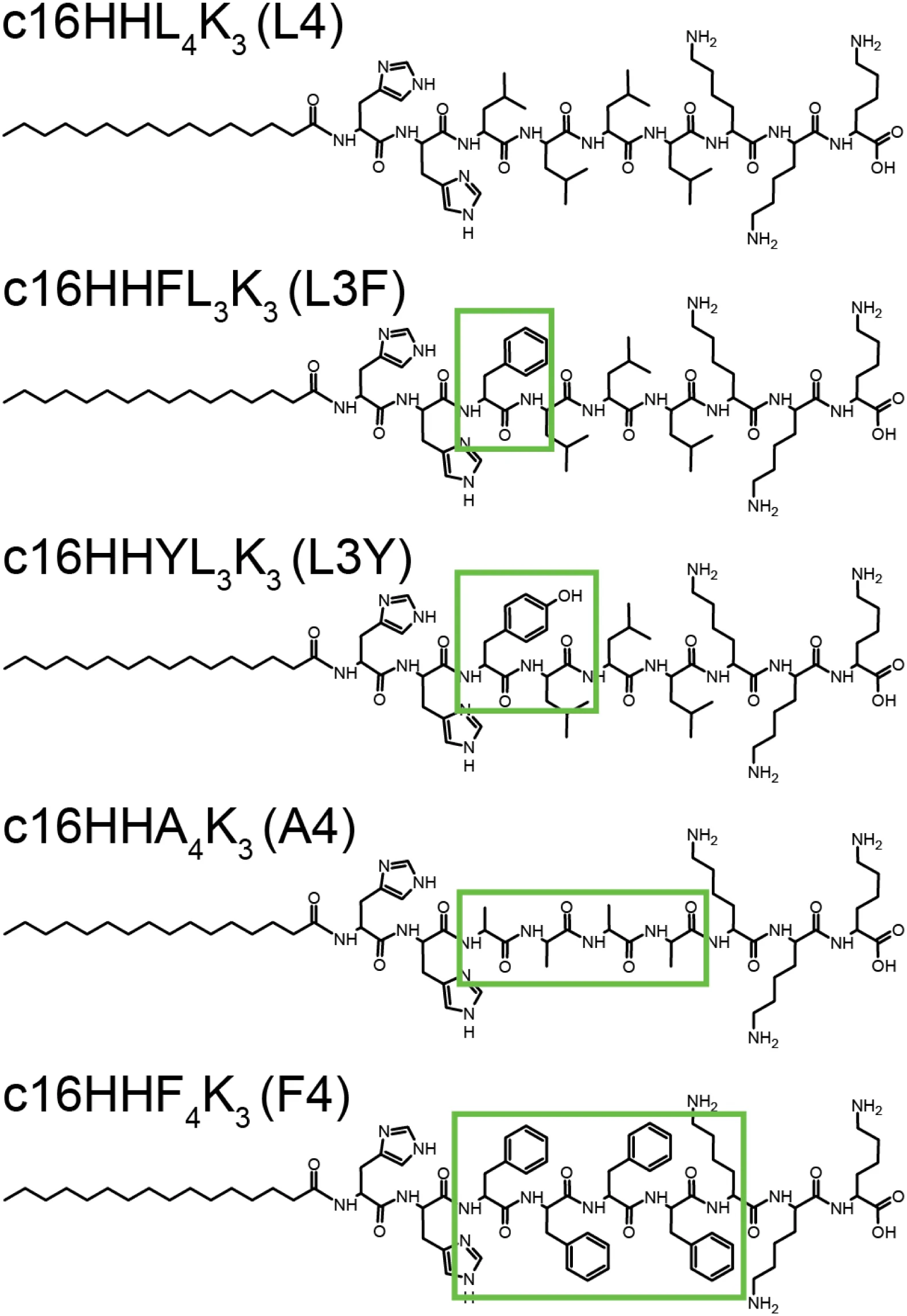
Chemical structures of the PAs used in these experiments. Inside the parentheses are the common names we use in this paper. Green boxes highlight the differences from our base peptide L4.

We first wanted to confirm that all PAs form fibers at pH 11, and used Atomic force microscopy (AFM) to visualize their structure. AFM confirmed that sequence variations did not inhibit the formation of robust, high-aspect-ratio nanofibers (Figure 4). All variants exhibited uniform cylindrical morphologies with diameters ranging from 6 to 10 nm, allowing us to attribute differences in redox stability solely to the chemical nature of the core residues rather than their macroscopic assembly.

**Figure 4:**
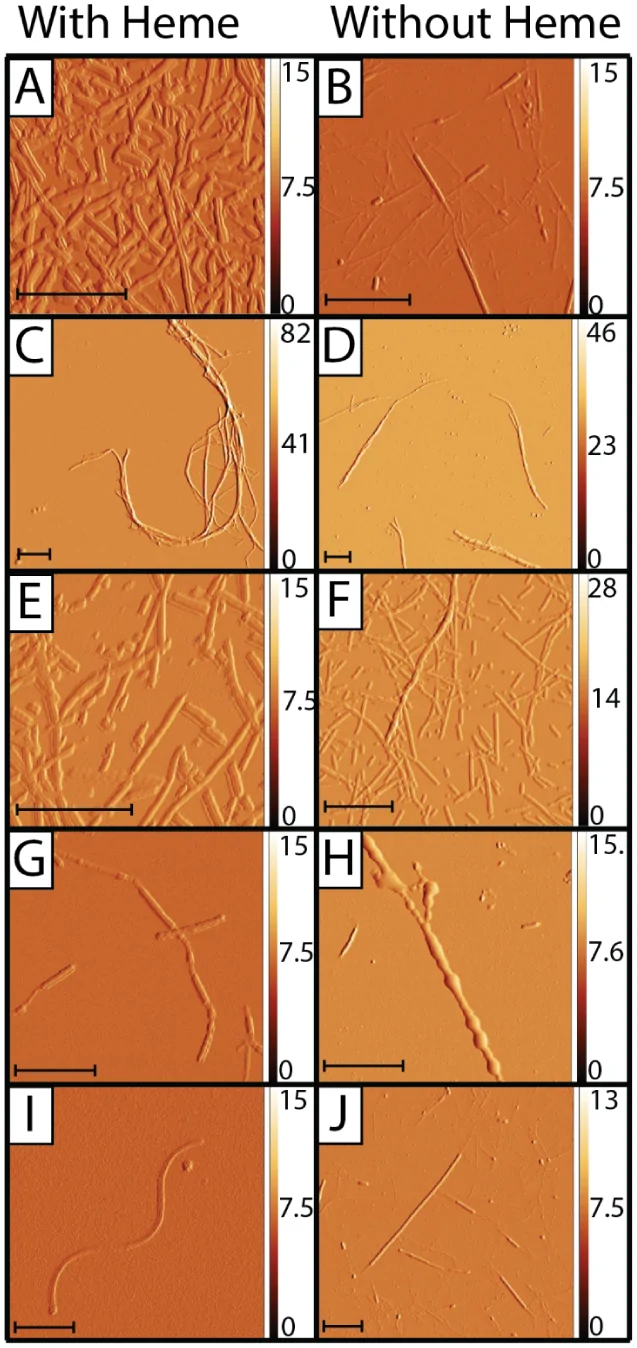
Atomic force microscopy (AFM) morphological characterization of peptide amphiphile (PA) assemblies. Height images of (A, B) L4, (C, D) L3F, (E, F) L3Y, (G, H) F4, and (I, J) A4 prepared in the absence and presence of encapsulated heme B. All variants exhibit the anticipated, high-aspect-ratio nanofibrillar morphologies at pH 11, demonstrating that supramolecular self-assembly is preserved upon cofactor incorporation. Samples were prepared in 15 mM NH_4_OH, deposited onto freshly cleaved mica, and dried prior to imaging. All scale bars represent 1 μm. Vertical color bars indicate topography height scales in nanometers.

While AFM imaging reveals varying degrees of lateral fiber bundling across the five peptide variants, we attribute these macro-structural variations primarily to evaporation-induced artifacts during sample drying on the mica substrate. To date, we have found no structural or literature evidence suggesting that such extensive bundling persists in aqueous solutions for these or related peptide amphiphile systems. Because all subsequent thermodynamic, spectroscopic, and kinetic experiments were conducted in aqueous media, we believe these dried-state macro-structures do not correlate with or influence the solution-phase redox behavior. This confirms that the observed kinetic protection is a strictly localized, sequence-dependent phenomenon rather than a macro-scale aggregation effect.

### Kinetics of Aerobic Oxidation and the Multi-Parameter Kinetic Model

To quantify the protective capacity of these synthetic scaffolds, we employed stopped-flow UV–vis spectroscopy to monitor the oxidation of prereduced fibers upon mixing with oxygen-saturated buffer at pH 11. Figure 5 tracks the disappearance of the ferrous Q-bands (556 nm) of reduced heme B (full spectra can be found in Figure S3). We observed that all five PAs exhibited slightly differing behavior. Immediately, we noticed that the L3F variant had the longest-lived reduced state of any PA tested, which we interpreted as having the strongest protection from solvent. F4 was able to protect the reduced heme, but not as strongly as L3F. L3Y had weak protection, however, it has a slow decay over time. L4 and A4 showed the shortest-lived reduced state which we interpreted as having weak protection against O_2_ in the solvent. Importantly, in the peptides L3F, F4, and L4 we observed a nonexpoential decay which warranted further analysis.

**Figure 5:**
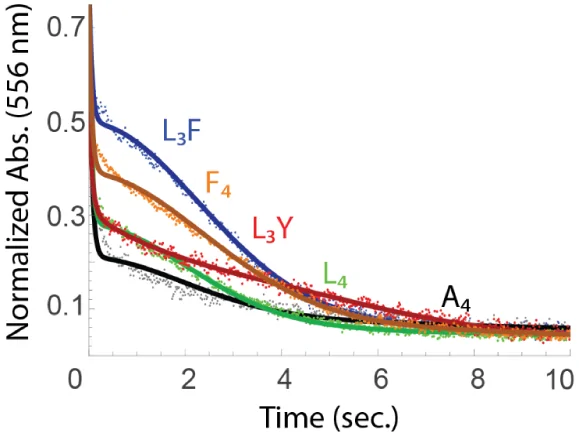
Oxidation kinetics of the five peptides tested. All samples were prepared with 50 μM heme and 300 μM PA. Temperature was set to 15 °C, samples are in 15 mM NH_4_OH, pH 11. Substoichiometric amounts of dithionite were added to reduce the samples. Fits were plotted with Mathematica software, version 14.3.

To evaluate the initial coordination environments of the encapsulated cofactors under exact operational conditions, the fully oxidized (ferric) spectra were extracted directly from the terminal end points of the time-resolved stopped-flow kinetic traces (Figure S4). Across all five peptide amphiphile variants (A4, L4, F4, L3F, and L3Y), the Soret maximum sit between 411 and 412 nm, which is highly characteristic of a standard, 6-coordinate low-spin bis-histidine ferric heme complexs. This demonstrates that despite variations in the steric bulk and hydrophobicity of the core side chains, each self-assembled matrix successfully achieves full axial bis-imidazole ligation *in situ*. Importantly, this rules out differences in initial coordination number or spin state as the origin of the differing oxidation kinetics, establishing that the exceptional kinetic protection unique to L3F must arise from another mechanism rather than static differences in the initial resting state.

The three PAs exhibiting complex, nonexponential decay traces could not be resolved by standard mono- or biexponential models. To quantitatively analyze these multiphase kinetics, the time-dependent absorbance traces were fitted to an 8-parameter multicomponent model (eq 2). This model integrates a Gaussian function to account for the initial temporal distribution of the protected heme population, followed by two discrete exponential terms representing the subsequent oxidation phases:

2 $$A_{x}=A_{1}e^{-k_{1}x}+A_{2}e^{-k_{2}x}+A_{B}e^{-0.5{\left(x-x0\right)}^{2}/\sigma^{2}}+C$$

Here, *A*_*B*_, *x0*, and σ define the Gaussian component. The subsequent biphasic exponential decay is characterized by amplitudes *A*_1_ and *A*_2_ and rate constants *k*_1_ and *k*_2_. A constant offset, *C*, accounts for baseline shifts. To mitigate the risk of overfitting inherent in an 8-parameter model, fits were validated by the randomness of the residuals (Figure S5) and the consistency of resolved parameters across independent replicates.

The Gaussian component accounts for a “protection phase”, an induction period where the tight packing of the L3F or F4 PAs create a high-energy barrier, representing a population of hemes that experience a distinct, nonfirst-order environment at the onset of the reaction. This is followed by a biphasic exponential decay which we attribute to the internal architecture of the PA fiber: the fast rate (*k*_1_) represents hemes located in solvent-permeable sites or at the fiber termini (end-caps), while the slow rate (*k*_2_) is the rate-limiting diffusion of O_2_ through the “locked” crystalline β-sheet interior. By resolving these three distinct populations, we can move beyond average rates and quantify the specific fraction of hemes (*A*_*B*_) that benefit from supramolecular insulation.

The subsequent biphasic decay provided a granular look at the internal fiber environment. Table 1 lists the extracted kinetic values. The fast rate constant *k*_1_ describes oxidation within disordered or solvent-accessible regions of the core, while the slow rate constant *k*_2_ represents the rate-limiting diffusion of oxygen through the crystalline β-sheet barrier. The L3F variant emerged as the most resilient, exhibiting an *A*_*B*_ of 0.41 and maintaining a reduced population for over five seconds. This suggests a synergistic effect where the leucine residues drive initial hydrophobic assembly while the phenylalanine side chains provide a bulky “gate” to obstruct oxygen entry. In contrast, the A4 variant, despite forming robust fibers, lacked the steric bulk to provide a significant kinetic barrier, leading to rapid oxidation across both exponential phases.

**Table 1:** Kinetic Parameters and Fitted Values from the Biexpoential-Plus-Gaussian Fit

Peptide	L4	L3F	L3Y	F4	A4
*k*_*1*_ (s^–1^)	18.0 ± 0.71	18.0 ± 1.24	15 ± 1.4	18.0 ± 0.81	18.0 ± 0.85
*A*_*1*_	0.48 ± 0.017	0.33 ± 0.021	0.24 ± 0.019	0.39 ± 0.016	0.61 ± 0.026
*k*_*2*_ (s^–1^)	0.089 ± 0.0032	0.080 ± 0.003	0.31 ± 0.0086	0.12 ± 0.0031	0.078 ± 0.0031
*A*_*2*_	0.055 ± 0.0016	0.068 ± 0.0019	0.26 ± 0.0066	0.089 ± 0.0028	0.071 ± 0.0019
*x0 (s)*	0 ± 0.060	0 ± 0.038	3.7 ± 0.17	0 ± 0.046	0 ± 0.16
σ (s)	2.03 ± 0.038	2.28 ± 0.025	2.6 ± 0.17	2.41 ± 0.031	2.0 ± 0.099
*A*_*B*_	0.20 ± 0.003	0.41 ± 0.0035	0.047 ± 0.0039	0.29 ± 0.0037	0.11 ± 0.0043
*C*	0.022 ± 0.0002	0.024 ± 0.0004	0.031 ± 0.0002	0.018 ± 0.0002	0.027 ± 0.00044

### Thermodynamic Evidence of Reorganization: Redox Hysteresis

We next wanted to test if the differences in reduced-heme stability come from the PA structure or from differences in the heme’s reduction potential. To do that, we ran potentiometric titrations of heme bound to our PA fibers. We observed mildly similar redox behavior and a striking degree of redox hysteresis for all five PA sequences (Figure 5, Table 2). Raw redox spectra for all five PA sequences can be found in Supplementary Figure 6. All five peptides show two sets of potentials depending on whether you start from the reduced or oxidized states of heme. While the values are not the same, we notice similar patterns for all PAs: the potential required to reduce the heme (E_mRed_) was significantly more positive than the potential observed during the subsequent oxidizing direction of this titration (E_mOx_). The magnitude of this hysteresis, termed ΔE_m_ also varied mildly in each peptide, ranging from 204 mV in L3Y to over 305 mV in the F4 variant. We note that the F4 PA has a higher E_mRed_and a lower E_mOx_than the other four PAs. We hypothesize this is due to the additional hydrophobicity and steric bulk of the Phe residues. However, that will be explored in future papers, as we believe it is beyond the scope of this present work.

**Table 2:** Reduction Midpoint Potentials for the PA Variants Tested in This Paper^a^

PA	E_mRed_	E_mOx_	ΔE_m_
**L4**	–141 ± 37.9	–358 ± 73.5	217
**L3F**	–142 ± 1.4	–372 ± 21.8	230
**L3Y**	–147 ± 20.1	–352 ± 31.2	204
**F4**	–106 ± 20.3	–411 ± 9.3	305
**A4**	–157 ± 29.5	–407 ± 5	249

a All values are reported against a 3M Ag/AgCl electrode, and all values are reported in mV.

In the context of supramolecular assemblies, the massive redox hysteresis (ΔE_m_ up to 305 mV) indicates a significant reorganization energy associated with the Fe^3+^/Fe^2+^ transition. This energy gap represents the structural work required to transition the peptide matrix from an open state to a closed, shielded state. This energy gap represents the structural work performed by the system to rearrange the PA monomers into a “locked” state, creating a barrier that must be overcome to return the system to its initial oxidized configuration. We do not see any pattern in the structure and E_m_ values to suggest any specific structural feature is responsible for the kinetic stability observed in the stopped flow data. All PAs appear to have similar behavior, with the slight exception of F4.^33^

### Structural Basis for the Supramolecular Gate

We then looked at the PA fiber’s structure to explain the differences in kinetic stability (Figure 6). We performed circular dichroism (CD) spectroscopy on the fibers in both their oxidized and reduced states (Figure 7). While all variants maintained a β-sheet signature, the structural response to the heme’s redox state was sequence-specific. For the L4 and L3F PAs, we notice that the spectral minima are 222 nm in both reduced and oxidized states, offset from the standard β-sheet signal of 216–218 nm. We attribute this to high levels of twisting imposed by the large fiber structures.^34^ The absence of a 208 nm shoulder confirms that this signal represents a highly twisted β-sheet rather than an α-helical transition. For all other peptides, the minima were in the standard 218 nm range, indicating classical β-sheets

**Figure 6:**
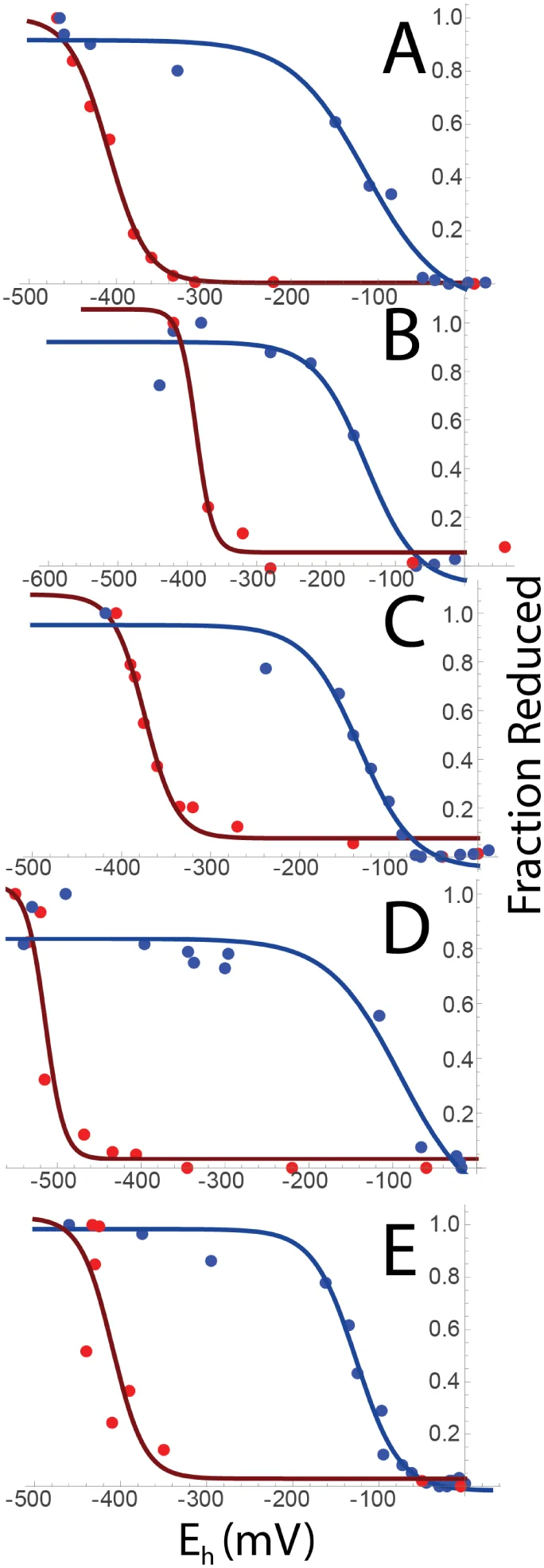
Redox potentiometric titrations for the PAs in this paper. (A) A4, (B) L3F, (C) L4, (D) L3Y, and (E) F4. All samples prepared as described it the Materials and Methods. E_h_ values measured using the 3 M Ag/AgCl electrode.

**Figure 7:**
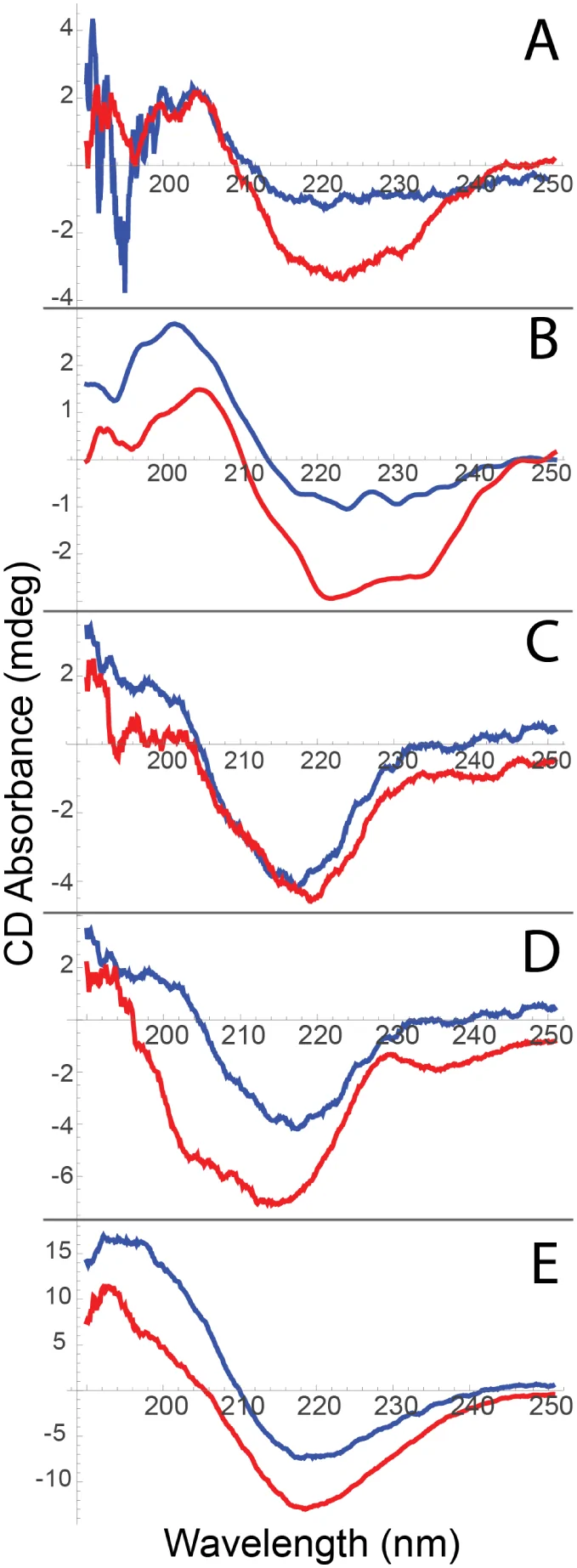
Circular dichroism spectra for the reduced (red) and oxidized heme (blue) in the PA fibers (A) L4, (B) L3F, (C) L3Y, (D) F4, and € A4. All samples were prepared as described in the Materials and Methods section. Data plotted using Mathematica 14.3.

The L3F variant exhibited the most pronounced response, with a significant increase in molar ellipticity at 222 nm upon reduction. This intensification is indicative of a cooperative structural tightening or an increase in the long-range order of the β-sheet scaffold. This redox-triggered conformational shift correlates directly with the kinetic protection observed in stopped-flow experiments, suggesting that the ferrous state facilitates a more crystalline, less permeable peptide shell.

Conversely, the L4 variant displayed a significant 2 nm red-shift but a decrease in overall signal magnitude. This indicates that while the leucine-rich core is responsive to the heme redox state, it fails to transition into the highly ordered, “locked” configuration necessary to exclude oxygen as effectively as L3F or F4. Interestingly, the A4 and L3Y variants showed minimal spectral changes. For L3Y, the presence of polar hydroxyl groups likely maintains a hydration shell, preventing the formation of a hydrophobic core. For A4, the lack of side-chain steric bulk results in a hydrated and exposed heme binding-site.

## Discussion

The development of synthetic electronic biomaterials that can operate in aerobic environments remains a formidable challenge.^9,35^ While natural proteins like neuroglobin and cytoglobin have evolved intricate protein folds to shield the heme cofactor from solvent-borne oxidants,^36,37^ recreating this protective environment within a self-assembled system requires precise control over both molecular packing and stimulus-responsive dynamics. Our results demonstrate that by tuning the primary sequence of peptide-amphiphiles, we observe a redox-triggered tightening, providing kinetic protection to the reduced heme B cofactor.

Our initial design rationale focused on the transition from mono-His to bis-His ligation. By incorporating two histidine residues (HH sequence), we observed a marked increase in the lifetime of the reduced heme compared to the HA variant (Figure 2). The significant increase in kinetic stability in HH variants could suggest a change in coordination geometry, which we hypothesize is the transition to a bis-histidine environment, which is supported by previous work.^24^ This creates a higher steric barrier to oxidation compared to HA controls.

The nonexponential oxidation kinetics observed in our most protective variants (L3F, F4, and L4) necessitated the use of an 8-parameter multicomponent model. This model’s inclusion of a Gaussian provides a quantitative metric for what we term the protection phase. In the L3F variant, the initial phase (defined by *x0* and σ) represents a temporal window where a subpopulation of the heme is almost entirely inaccessible to oxygen.

For the L3Y variant, the resolved Gaussian center (*x0* ≈ 3.7 s) and low amplitude (*A*_*B*_ = 0.047) indicate a poorly defined protection phase that is mathematically distinct from the *x0* ≈ 0 s observed in L3F. We attribute this to the high width (σ) and low *A*_*B*_, which suggest that any potential kinetic shielding is spatially and temporally ill-defined. Physically, this supports the hypothesis that the polar hydroxyl group of tyrosine prevents the formation of a strictly anhydrous, protected core, resulting in a structural environment that cannot effectively exclude oxygen at the onset of the reaction. Consequently, the L3Y data demonstrates that steric bulk alone is insufficient for air-stability; a strictly hydrophobic dielectric environment is required to trigger the structural tightening observed in the CD spectra.

We observe a unique kinetic profile for the L3Y variant, it has the lowest *k*_1_ paired with the highest *k*_2_ across the nonprotective sequences. We attribute this distinct behavior to the structural consequences of the phenolic hydroxyl group on the tyrosine residue buried within the fiber core. In the resting reduced state, we hypothesize that this localized polarity organizes a highly stable, “locked” network of structural water molecules and interstrand hydrogen bonds. This rigid subnetwork imposes an initial kinetic barrier that suppresses dynamic peptide fluctuations, effectively delaying initial oxygen penetration and resulting in a minimized *k*_1_. However, once the first wave of oxidation occurs, the accompanying structural reorganization disrupts this delicate hydrogen-bonding network. Deprived of its cooperative stabilization, the local fiber architecture collapses into a disordered core state. This rapid structural breakdown opens hydrophilic conduits that expose the remaining ferrous centers to the solvent, leading to the characteristically rapid secondary oxidation phase (*k*_2_).

We propose that the differences in the “fast” (*k*_1_) and “slow” (*k*_2_) rate constants reflect the internal heterogeneity of the nanofiber. The *k*_1_ phase likely represents the oxidation of hemes located at the “ends” of the fibers or within disordered segments where the β-sheet packing is frustrated. Conversely, the *k*_2_ rate represents oxygen diffusion through the crystalline, “locked” interior of the fiber. The fact that L3F exhibits an *A*_*B*_ of 0.41 suggests that the introduction of a single phenylalanine residue creates a steric “bottleneck.” Unlike the A4 variant, which lacks sufficient bulk to exclude solvent, or the F4 variant, which may suffer from overpacking that prevents a clean transition, the L3F design balances leucine-driven assembly with phenylalanine-mediated protection.

A defining feature of these assemblies is the significant redox hysteresis observed in the potentiometric titrations (Figure 6, Table 2). In a standard, solvent-exposed heme system, the reduction and oxidation potentials should be nearly identical (i.e., E_MRed_ ≈ E_MOx_). However, our PAs exhibit a ΔE_M_ ranging from 204 to 305 mV. In the context of supramolecular chemistry, this hysteresis is a thermodynamic signature of structural reorganization. While the more positive E_MRed_ of F4 suggests a higher thermodynamic driving force for reduction, its slower oxidation kinetics compared to A4 highlight that the kinetic barrier imposed by the permeability of the reorganized matrix dominates over the electronic driving force in these assemblies.

The massive redox hysteresis observed (ΔE_M_ up to 305 mV) serves as a functional signature of a redox-coupled structural transition. While the magnitude of this gap may include kinetic contributions due to the semicrystalline nature of the fibers, it provides a quantitative measure of the effective energy barrier protecting the ferrous center. The 5–10 min equilibration time at each potential step was insufficient to revert the “locked” state in the F4 variant, indicating that the transition from a shielded reduced core to a porous oxidized shell is significantly hindered. This “conformational trapping” ensures that once the heme is reduced, the surrounding phenylalanine/leucine core tightens, which is supported by the intensification of the 222 nm signal in the CD spectra. Thus, the 305 mV gap represents the “mechanistic cost” of unlocking the supramolecular gate.

Looking to the Circular Dichroism (CD) data, the increase in the β-sheet signal for L3F upon reduction is evidence of cooperative tightening of the peptide scaffold. We propose that the transition from Fe^3+^ to Fe^2+^ alters the charge density and coordination geometry of the heme, which in turn allows β-sheets in the PA fiber to twist into a conformation with a less accessible cofactor. In the oxidized state, the fiber is relatively “relaxed” or “porous,” allowing hemes to be reduced by an external reductant (high solvent accessibility). However, upon reduction, the heme iron becomes Fe^2+^, triggering the peptide monomers to twist and pack more tightly (as seen in the 222 nm CD shift). In this protected state, the fiber excludes O_2_ kinetically, creating the Gaussian protection phase observed in the stopped-flow experiments.

In contrast, the L4 variant, which shows a decrease in CD magnitude upon reduction, undergoes a conformational shift that fails to increase crystallinity. Without the phenylalanine residue, the leucine core rearranges but does not have sufficient steric bulk to prevent rapid oxidation of the heme.

This raises the critical question of whether the observed kinetic protection is strictly sequence-specific or secondary-structure-specific. Circular dichroism (CD) spectroscopy confirms that all five peptide amphiphiles adopt robust, highly similar β-sheet secondary structures in their resting states. Furthermore, the change in the β-sheet signal upon reduction (Δβ-sheet) is remarkably similar between the highly protective L3F variant and the nonprotective L4 control. This decoupling between global secondary structure reorganization and kinetic protection is highly revealing. It demonstrates that while a redox-triggered contraction of the β-sheet backbone framework (Δβ-sheet) is a necessary global prerequisite, it is ultimately insufficient on its own to insulate the cofactor. Instead, the local identity of the side chains within that contracted framework dictates the sealing of the pocket. In the case of L4, global framework tightening merely packs uniform leucine residues tighter, which fails to provide an absolute steric barrier to oxygen. For L3F, however, that identical global backbone contraction positions the bulky, aromatic phenylalanine side chains into an interlocking, high-density steric arrangement that specifically seals the local binding pocket. Thus, the macro-structural framework is secondary-structure-driven, but the dynamic gating mechanism itself is fundamentally sequence-specific.

Our findings indicate that the kinetic stability of reduced heme within supramolecular PAs is not merely a static property of the assembly, but is governed by sequence-dependent reorganization energy and redox-triggered conformational dynamics. By systematically modulating side-chain hydrophobicity and steric bulk, we have demonstrated that a single phenylalanine residue (L3F) can create a significant kinetic barrier to oxygen diffusion, effectively shielding the ferrous center for several seconds under ambient aerobic conditions.

This “gating” mechanism offers a robust strategy for the design of air-stable bioelectronic materials. Rather than relying on bulky protein scaffolds or anaerobic environments, these minimal 9-residue and a palmitic acid PAs provide a programmable dielectric environment that can tune the kinetic stability of the embedded reduced cofactors. Such scaffolds could serve as “insulated” conduits for long-range electron transport (LRET), where the peptide matrix suppresses off-pathway electron leakage to ambient oxygen, a primary challenge for current synthetic organic semiconductors and microbial-inspired nanowires.

Furthermore, the significant redox hysteresis and conformational “tightening” observed in the L3F and F4 variants identify these assemblies as ideal bottom-up model systems for deconstructing the design rules of natural multiheme cytochromes, such as OmcS and MtrCAB. Because the electrochemical profiles of these PAs align closely with the reduction potentials of microbial cofactors involved in extracellular electron transfer, they provide a modular platform to investigate how specific aromatic stacking and hydrophobic burial dictate the efficiency and directionality of electron flow. Ultimately, utilizing these principles to design PAs that protect sensitive redox centers allows for the development of biointerfaced electrodes and diagnostic sensors that maintain electrochemical integrity in oxygenated physiological fluids.

Ultimately, the divergent insulation capabilities across this peptide series highlight a fundamental design principle in de novo protein engineering: while a stable secondary structure framework is required to assemble the active matrix, the functional output is governed by localized, sequence-specific packing dynamics. This mechanism is conceptually summarized in Figure 8. Under exact operational conditions, all five variants initiate from a spectroscopically uniform, 6-coordinate low-spin bis-histidine resting state, establishing that the baseline assembly architectures are identical. Upon reduction, however, global framework contraction drives a mechanical divergence. In the asymmetric L3F core, a compliant leucine matrix allows the singular, bulky phenylalanine plug to cooperatively seal the remaining solvent channels, resulting in exceptional kinetic protection. Conversely, uniform homopolymeric frameworks either fail to construct a localized steric barrier (L4/A4) or remain locked in an unyielding, statically rigid aromatic cage (F4) that permits steady baseline leakage. Peptide design strategies must therefore look beyond static secondary-structure stability and intentionally program localized steric asymmetries to gate cooperative macromolecular functions

**Figure 8:**
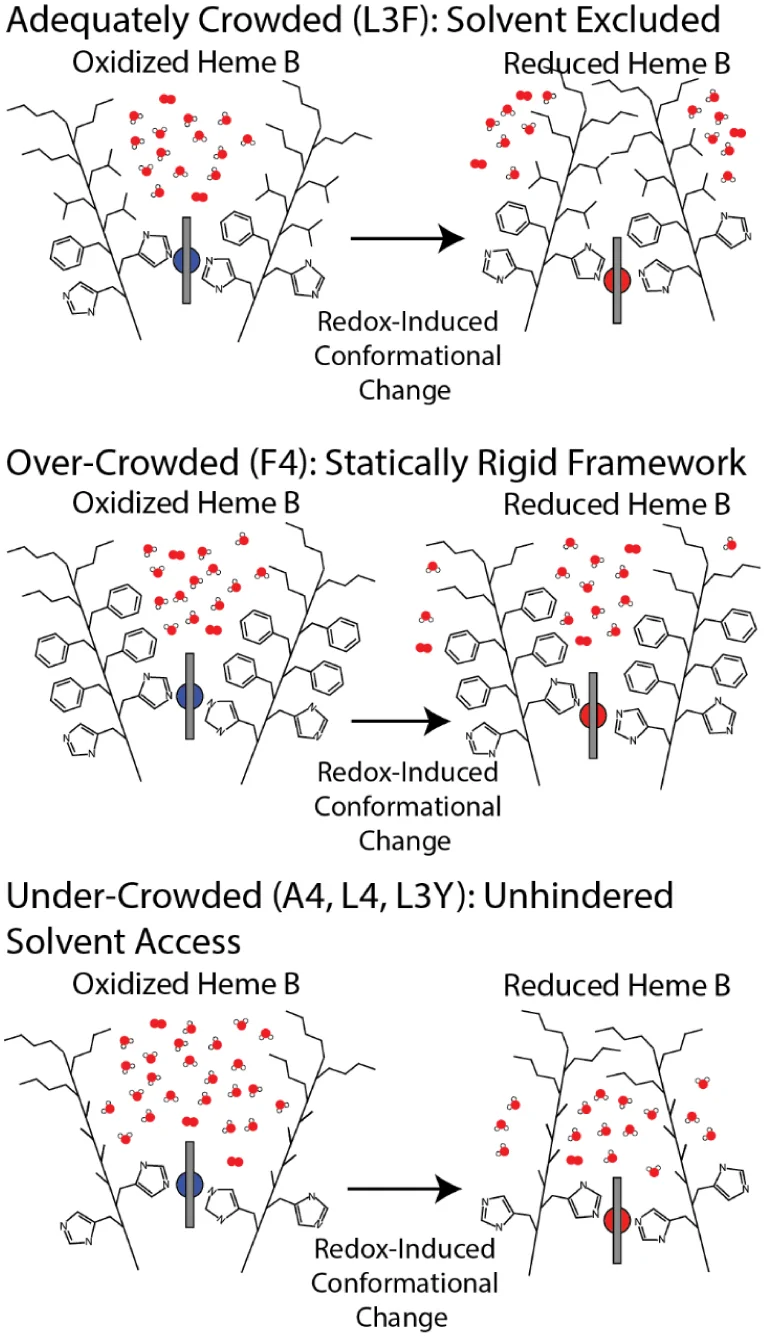
Schematic of redox-triggered supramolecular gating. Left column depicts the bis-histidine-ligated oxidized (Fe^3+^) resting state across all PAs in this work. Right column illustrates the sequence-specific structural divergence that occurs upon reduction (Fe^2+^). In the asymmetric L3F core (top), leucines and the bulky phenylalanine side chain pack into an interlocking arrangement, sterically sealing the heme cofactor away from solvent and oxygen. In contrast, the overcrowded F4 framework (middle) is trapped in a statically rigid aromatic cage that cannot collapse or clamp, while the under-crowded L4/A4 frameworks (bottom) contract without establishing a localized steric barrier, leaving the heme center highly solvent-accessible.

## Conclusions

By treating the supramolecular nanofiber as a dynamic, stimulus-responsive material rather than a static scaffold, we have demonstrated a robust strategy for protecting reactive redox centers under aerobic conditions. Our systematic investigation of the c16HHX4K3 series identifies the L3F variant as a “Goldilocks” design that balances the competing requirements of assembly and protection. This specific sequence provides sufficient hydrophobicity to drive initial self-assembly, enough steric bulk to create a kinetic gate, and the requisite structural flexibility to undergo a redox-triggered tightening. Ultimately, this work bridges the gap between the complex evolved protection mechanisms of natural cytochromes and the modular simplicity of de novo supramolecular materials, enabling advanced functionality in real-world atmospheric environments.

Based on the structural, thermodynamic, and kinetic insights gathered across this series, we can define three explicit design rules to guide the development of next-generation insulated supramolecular redox biomaterials:

The first rule describes the asymmetric “Goldilocks” core packing: Total substitution of the core with maximal aromatic bulk (e.g., the F4 variant) creates a rigid, overpacked matrix with high thermodynamic hysteresis but poor kinetic protection, as the matrix cannot flexibly rearrange around the cofactor. Conversely, nesting a single bulky aromatic residue within a more compliant hydrophobic matrix (L3F) provides the optimal balance of structural flexibility and localized steric density necessary to cooperatively “clamp” and seal the binding pocket.

The second rule concerns strict exclusion of polar functional tethers. To achieve long-lived kinetic protection, the microenvironment immediately surrounding the cofactor must maintain absolute hydrophobicity. As demonstrated by the L3Y variant, introducing even a single polar group (−OH) creates localized hydrophilic pathways or stabilizes trapped structural water networks, effectively dismantling the anhydrous seal and accelerating baseline leakage.

Finally, the third rule describes the coupled framework twisting as a screening metric. True kinetic protection requires an explicit mechanical coupling between electron transfer and matrix reorganization. Future material designs can be effectively screened via redox-dependent CD spectroscopy; sequence variants are only viable candidates if reduction drives a cooperative increase in superhelical twisting or twisted β-sheet crystallinity (e.g., the negative 222 nm amplitude shift), rather than a disordered matrix relaxation.

Application of these design principles will enable the rational engineering of air-stable, peptide-based bioelectronic frameworks and synthetic metallonanofibers with precisely tuned redox lifetimes

## Supplementary Material



## Notes

Peptide amphiphile HPLC and mass spectrometry data; stopped-flow spectra; residuals for the biexponential plus Gaussian fits; redox titration spectra (PDF)

## #

Uniformed Services University School of Medicine, Bethesda, MD, 20814

## Abbreviations

CCR2: CC chemokine receptor 2
CCL2: CC chemokine ligand 2
CCR5: CC chemokine receptor 5
TLC: thin-layer chromatography
