# Supplementary material for: Quantifying the Sequence-Dependent Kinetic Protection of Reduced Heme in Peptide Amphiphile Nanofibers
Source: J Phys Chem B. 2026 Aug 10;130(33):8423–33. doi: 10.1021/acs.jpcb.6c02307 (PMC13501876; doi:10.1021/acs.jpcb.6c02307)
Supplement: Supplementary file 1 [file jp6c02307_si_001.pdf]

Quantifying the Sequence-Dependent Kinetic Protection of Reduced Heme in Peptide Amphiphile Nanofibers

Blake Campbell,[1$] Abigail Rogers,[1] Nathan A. Hernandez-Martinez,[1] Subas Dangi,[2] and Lee A. Solomon*[2].

AUTHOR ADDRESS (Word Style "BC_Author_Address"). [1]Department of Biology, and [2]Department of Chemistry, George Mason University, Fairfax, VA 22030, United States of America

KEYWORDS (Word Style "BG_Keywords"). Peptide Amphiphiles, Heme Reduction, Redox Kinetics

Table of Contents

Figure S1: HPLC Traces for the Peptide Amphiphiles discussed in this paper……..……………………..S1
Figure S2: MALDI Mass Spectrometry Data for the Peptide Amphiphiles discussed in this paper….S2
Figure S3: Stopped flow spectra for the peptide amphiphiles exposed to oxygen……………………….S3
Figure S4: UV Vis Spectra of Heme-Bound Peptide Amphiphiles…………………………………………..S4
Figure S4: Residuals for the biexpoential plus gaussian fits…………………………………………………S5
Figure S5: Representative Redox Titration spectral data…………………………………………………………S6

Supplemental Material

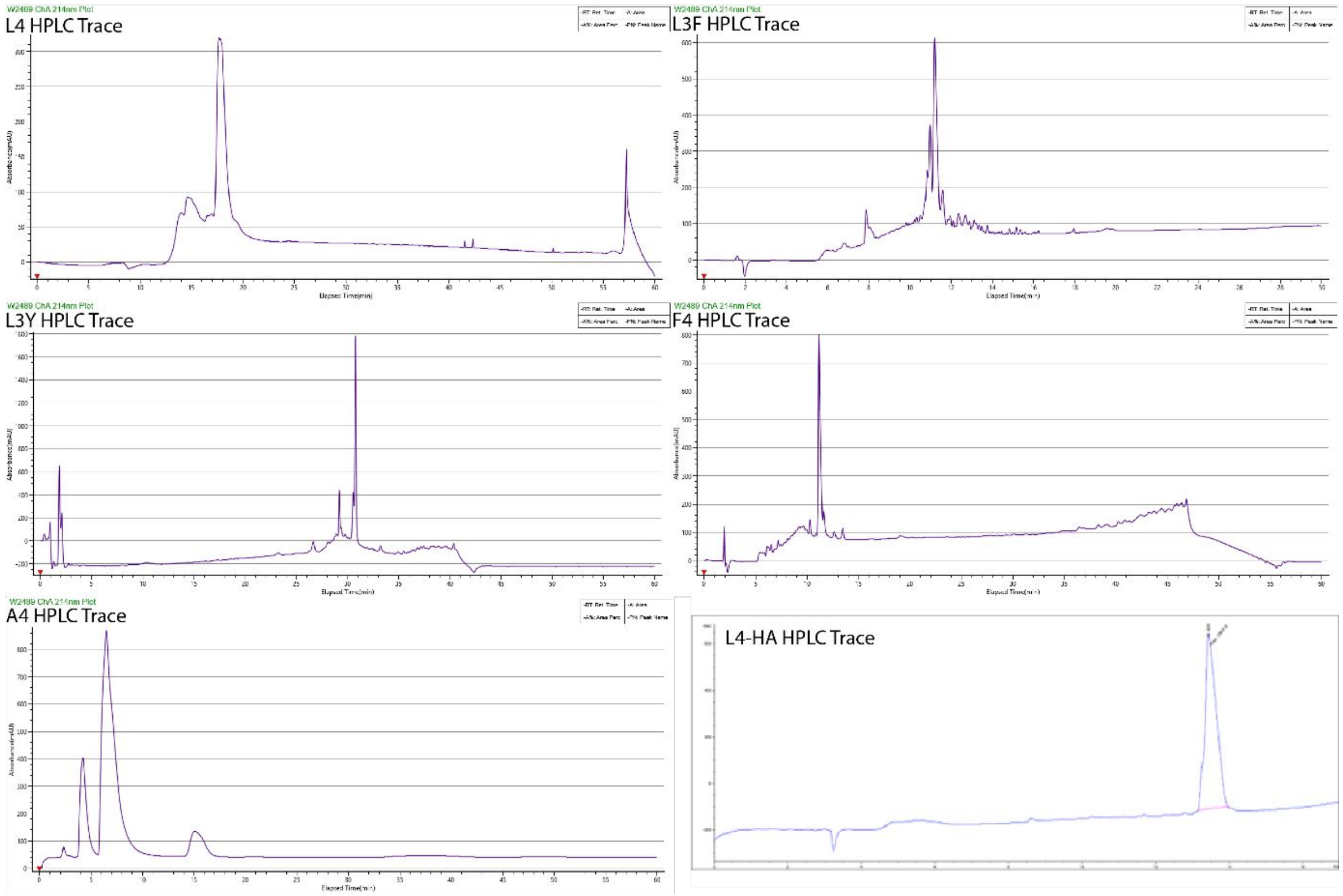

Figure S1: HPLC Traces of the peptide amphiphiles used in this paper.

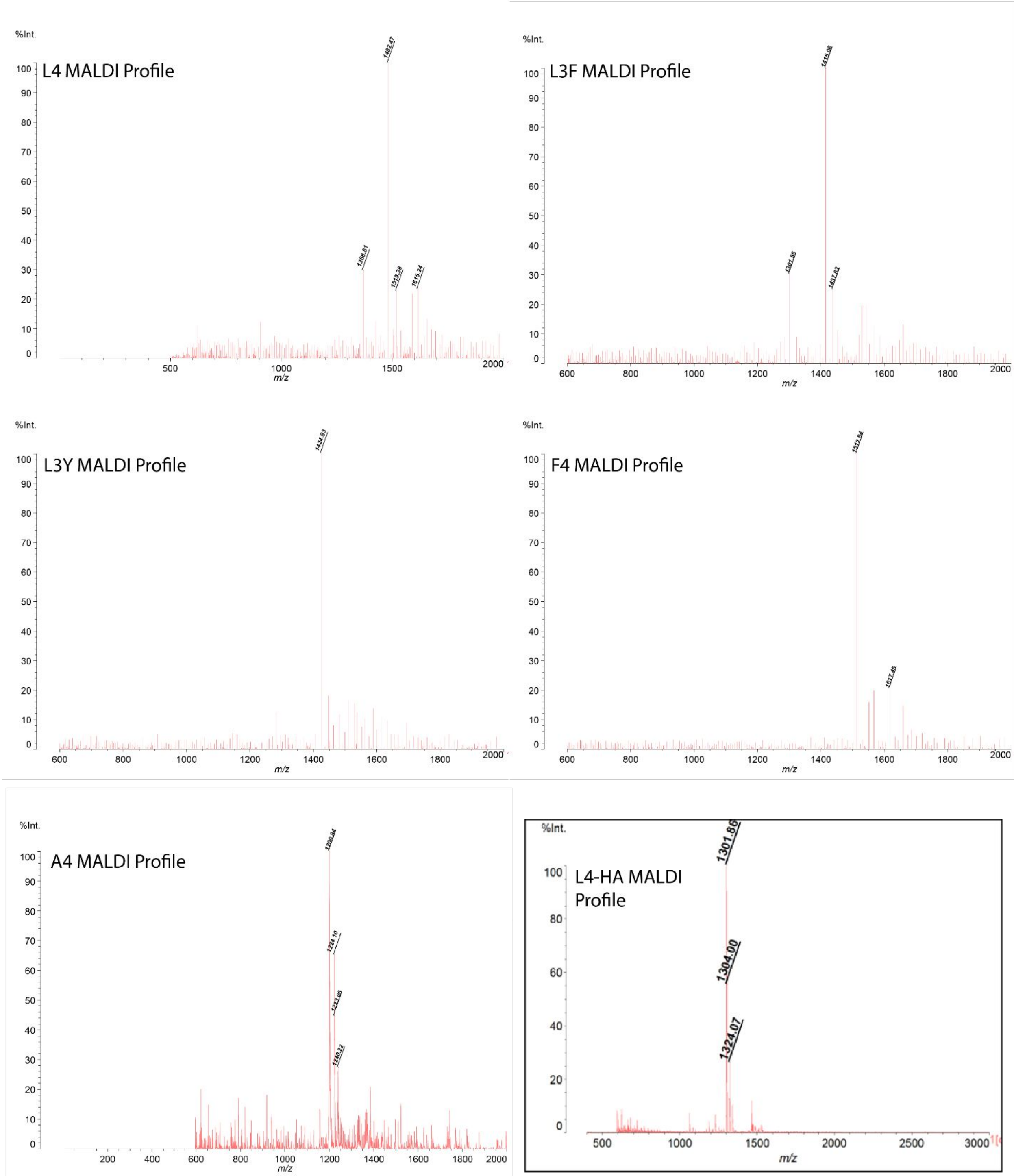

Figure S2: Matrix Assisted Laser Desorption/Ionization (MALDI) Mass Spectrometer profiles for the peptide amphiphiles used in this work.

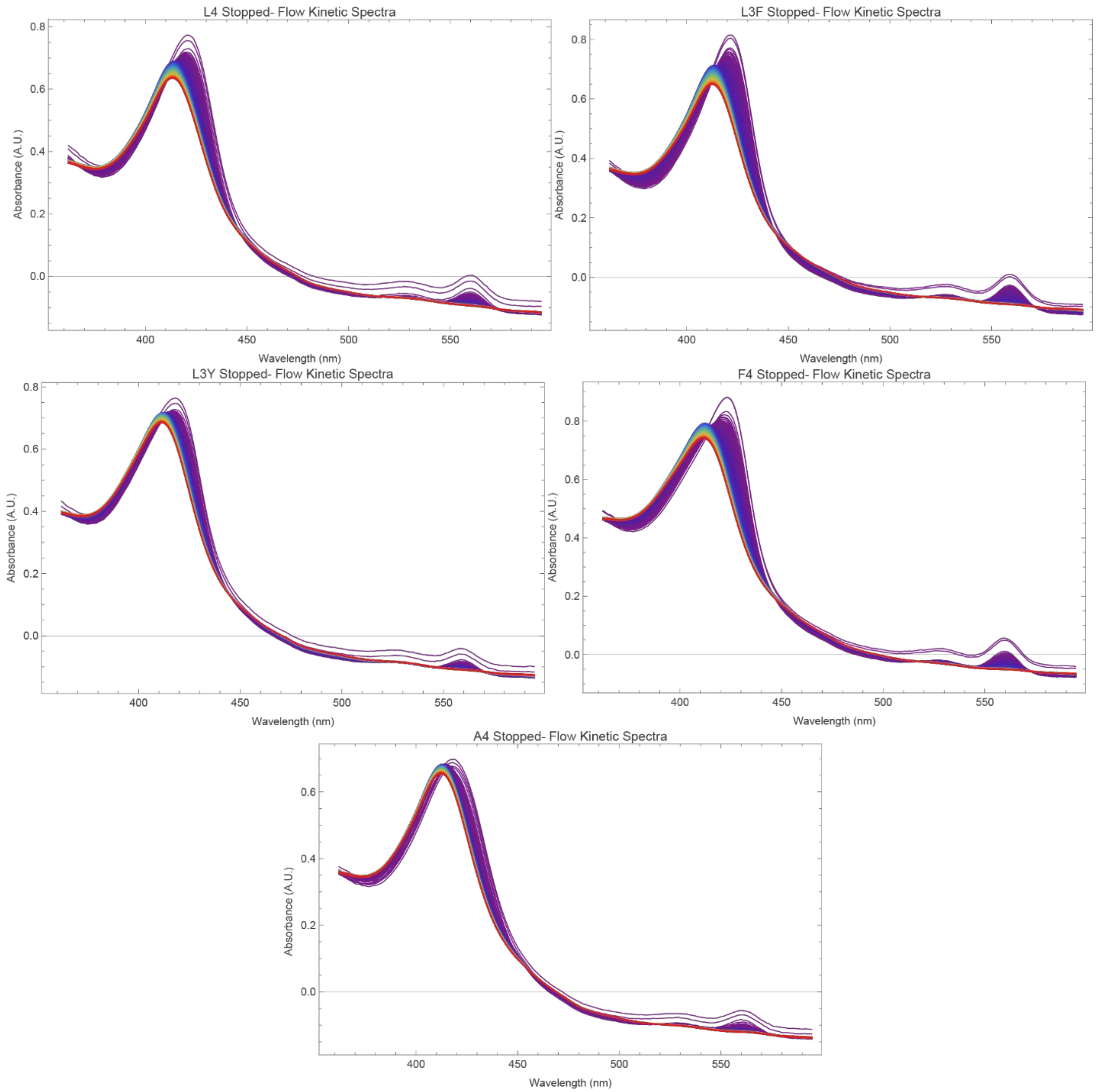

Figure S3: Raw Stopped flow spectra of all the peptide amphiphiles used in this work

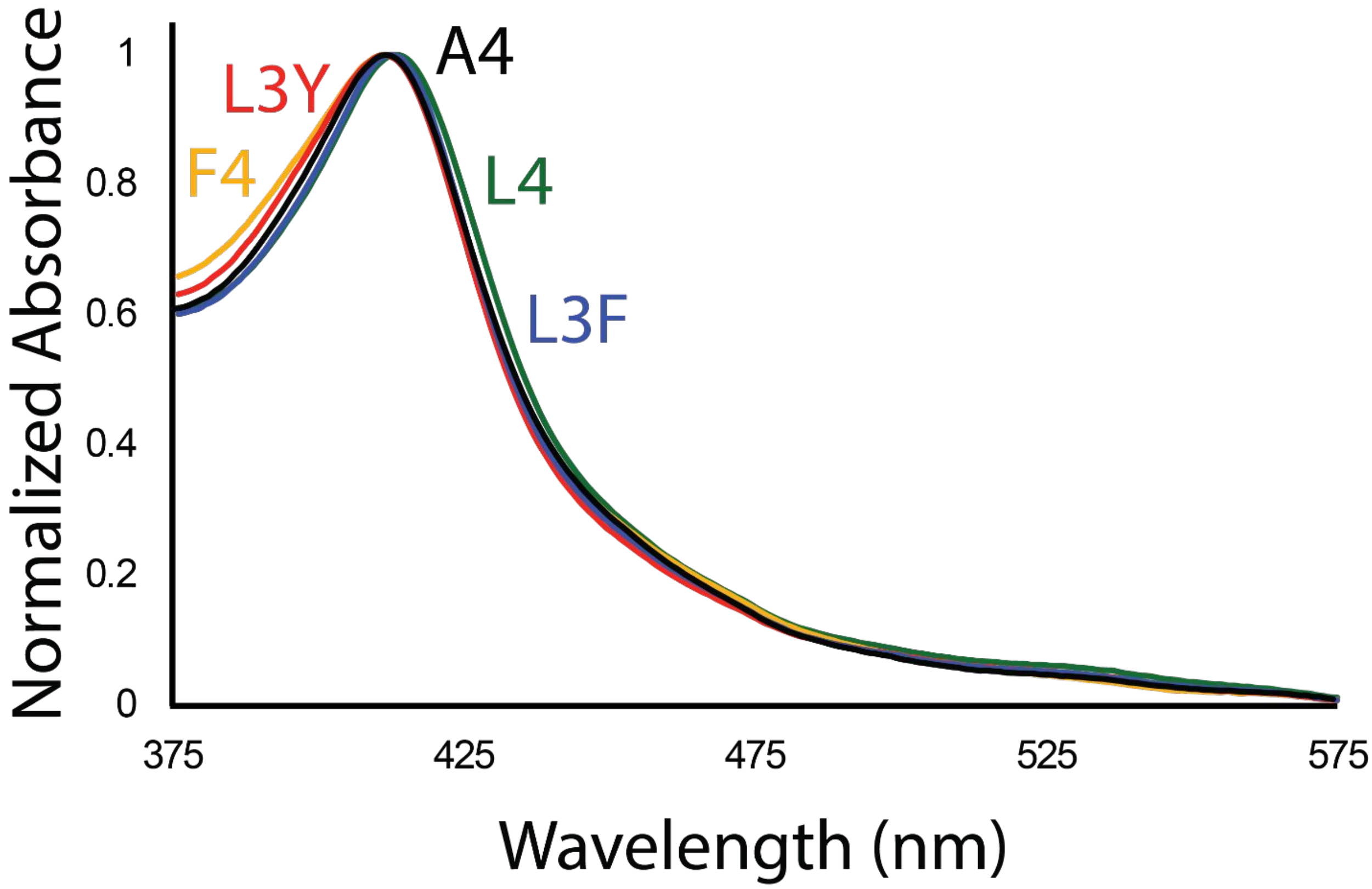

Figure S4: Normalized UV Vis spectra of heme B bound to fibrous assemblies of the five PAs used in this paper.

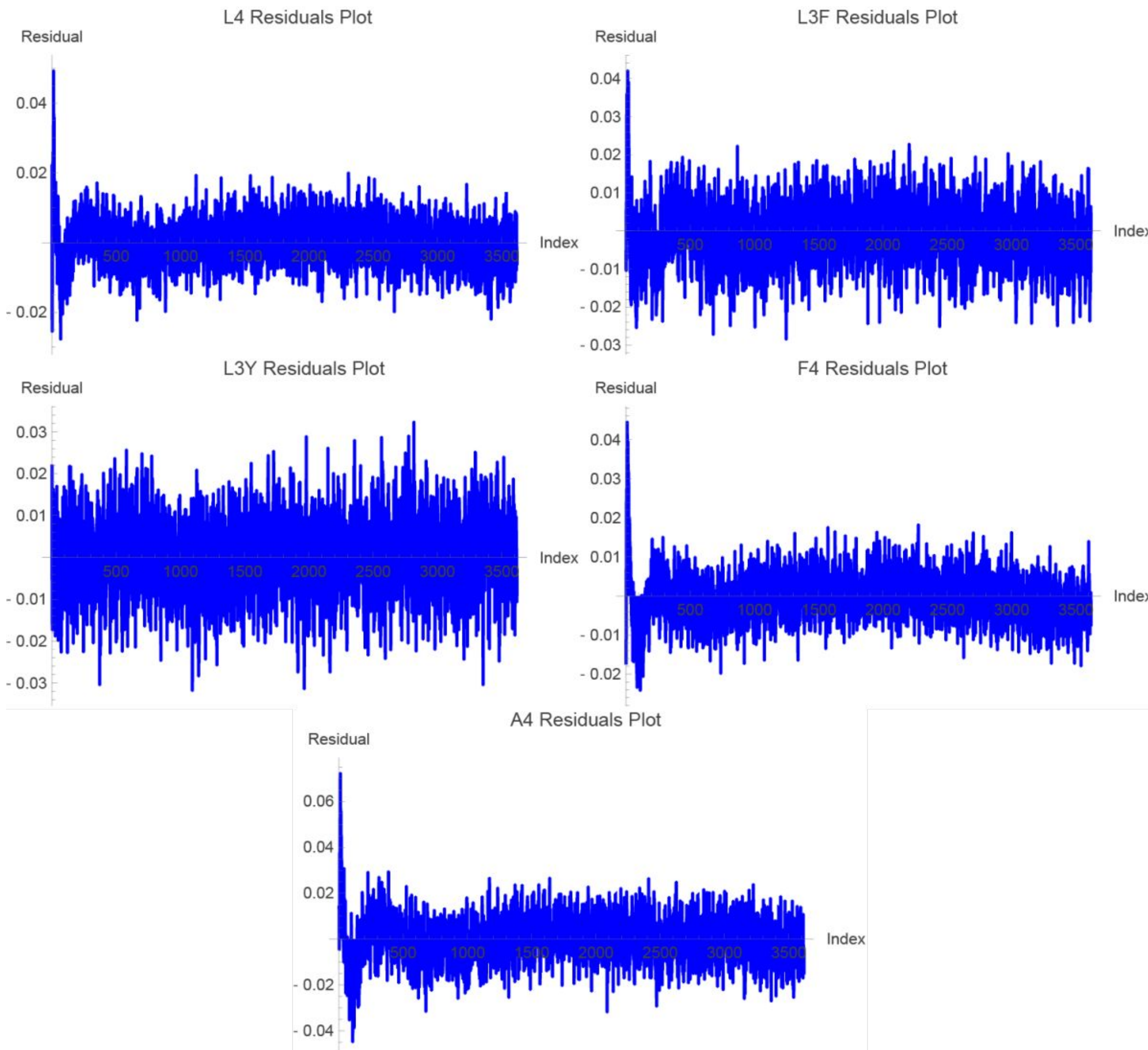

Figure S5: Residual fit data for all of the peptide amphiphiles used in this work. Residuals were plotted in Mathematica 14.3

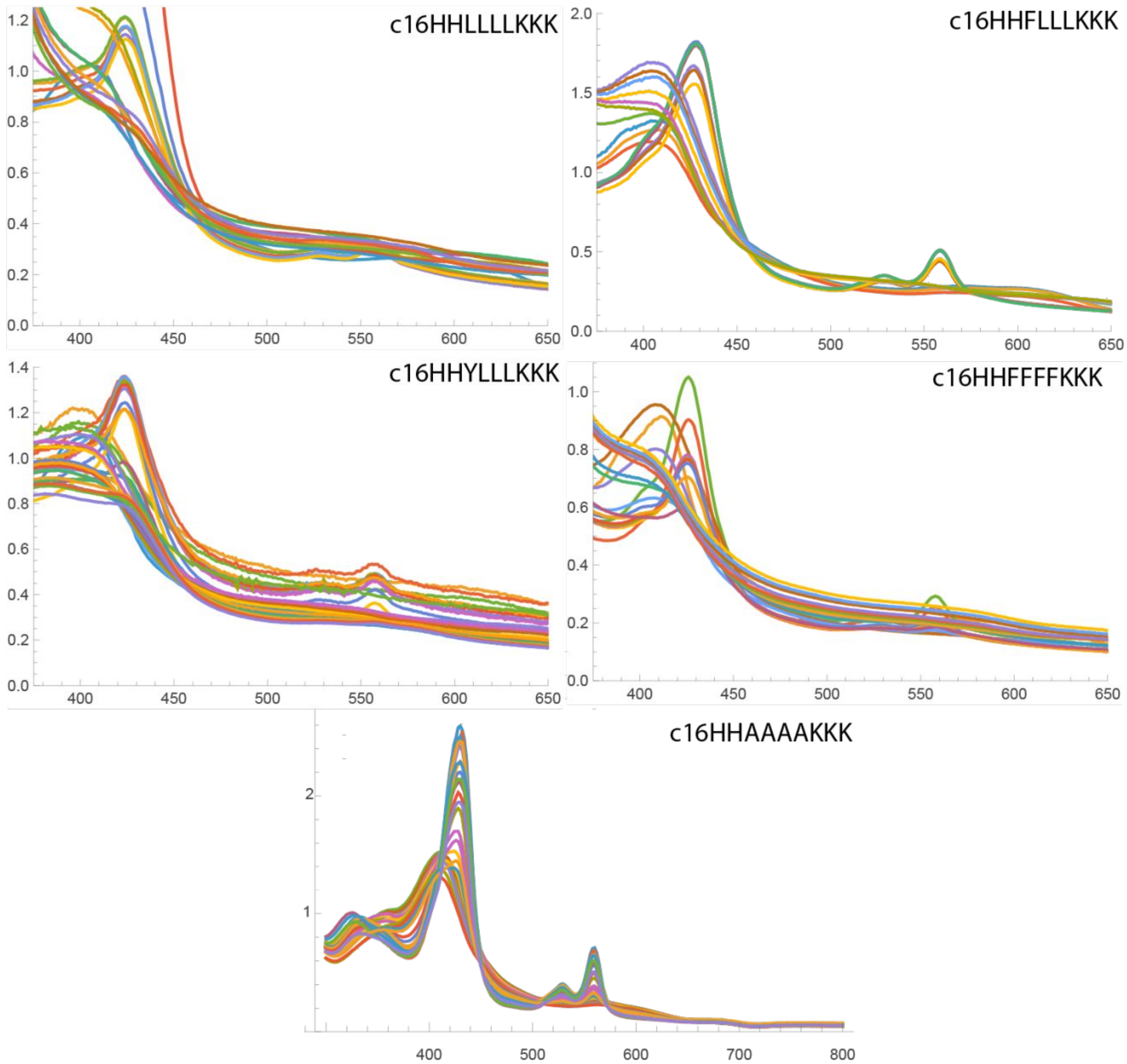

Figure S6: Representative raw redox titration data for all of the peptide amphiphiles used in this work.
